# The Knowledge Creation and Transfer Mechanism

**DOI:** 10.1007/978-3-030-62662-4_8

**Published:** 2020-11-24

**Authors:** Louise Ackers, Gavin Ackers-Johnson, Joanne Welsh, Daniel Kibombo, Samuel Opio

**Affiliations:** 6 Global Social Justice, University of Salford, Salford, UK; 7 University of Salford, Salford, UK; 8 Infectious Disease Institute, Kampala, Uganda; 9 Pharmaceutical Society of Uganda, Kampala, Uganda

**Keywords:** Knowledge creation, Knowledge mobilisation, Continuing medical education, Co-working, Co-presence, Mentoring

## Abstract

This chapter reflects on the relationship between the knowledge mobilisation processes that have contributed to behaviour change at an individual and organisational level. It critiques the traditional emphasis in international development on one-off, formal, foreign-led ‘training’ episodes and contrasts these with the more fluid, bilateral, approach to learning through co-working and mentoring.

Chapter 10.1007/978-3-030-62662-4_1 noted the quite prescriptive approach taken, by the funding partners, to knowledge mobilisation processes and associated log-frame audit. This chapter examines some of the underlying mechanisms that have shaped behaviour change in the MSI intervention. Knowledge for Change has been working to support health systems change in Uganda for a decade. This has involved huge organisational learning which we often describe as learning from failure. Rajkotia similarly argues that, ‘the fear of failure, instilled by the success cartel is one of the key reasons for why there is so little innovation in the health development sector arguing that ‘intelligent failures arising from experimentation and exploration are ‘praiseworthy’ (2018: 2). Our first book, ‘Killing me Softly’ (Ackers and Ackers-Johnson 2016) describes the challenges involved in foreign engagement to support systems change in Uganda’s public health system. Through a wide variety of projects, we have tried, with varying degrees of success, to work collaboratively with an emphasis on medium-long term sustainability. The book described what we have come to identify as a key weakness in approaches to foreign engagement, especially in short-term externally funded projects, namely a tendency to identify the ‘problem’ as one of the knowledge deficits and attempt to solve that through fly-in-fly-out short courses delivered by foreign ‘experts’.

Not only has this been seen to fail as a knowledge transfer mechanism; emphasising explicit (scientific or clinical) knowledge to the detriment of more implicit implementation knowledge is unlikely to deliver sustained behaviour change. A briefing document by Jhpiego,^1^ an affiliate of John Hopkins University reports that, ‘countries and donors spend significant amounts of funding on in-service training’ and yet, ‘traditional training approaches that focus on extended, off-site, group-based workshops have had limited effectiveness in improving and maintaining provider performance after training’. The brief cites a systematic literature review of interventions to improve healthcare provider performance in LMICs (Rowe et al. 2019) which found that ‘one-time training interventions results in very low effect size’. Indeed, they report learning outcomes of this kind of training as ‘low to none’. The approach specified by Jhpiego involves the use of ‘low dose, high frequency’ training. Rowe further contends that (any form of) training alone is not sufficient to improve quality; ‘when training is combined with Quality Improvement efforts, such as coaching or supervision, the effect size is significantly greater’.

Building on our mentoring approach, we have applied this through what we have termed, ‘bite-size, bedside’ reciprocal learning. The emphasis on formal training in LMICs (CMEs) has also generated unintended consequences associated with encouraging absenteeism and per diemism. The endemic expectation that staff will be paid for training has a poisonous effect on everything foreign organisations and volunteers try to do. In the course of this intervention, this expectation raised its head on many occasions. The following is a typical response to the proposal to organise a CME. K4C has always provided refreshments for meetings but never pays per diems. However, when we proposed to run IPC training, with attention to COVID-19 preparedness, we were expected to pay staff to attend:

People were looking forward to being paid even after telling them this is informal…

The normalisation of this expectation and the contribution such payments make to livelihoods resulted in a Ugandan colleague suggesting that we consider making financial payments in place of refreshments. The extent to which this ‘poison’ has begun to pollute even mentoring relationships is evident in the following conversation involving a senior doctor, who is generally absent from the ward, pressurising a Ugandan K4C health worker for money:

He is saying that he is giving a lot of support to (highly experienced UK doctor) and therefore he needs some kind of pay for this work.

The use of per diems has commodified and distorted incentives for training. It often results in the wrong staff (often only senior or managerial staff) being trained and pollutes team relationships. Our previous book reviewed the effectiveness of these approaches in anything but the immediate term (where pre- and post-test results, as anticipated in log-frame approaches, may show knowledge acquisition) when it comes to understanding behaviour change dynamics. As much behaviour change theory^2^ argues, adding knowledge alone (to increase individual ‘capabilities’) will rarely work when the opportunities to exercise that knowledge are missing. This is particularly evident when it comes to formal training on hand hygiene, for example, when—as was the case on the PNG ward—there is no running water, soap, towels or hand gel. In such situations, training staff in standardised international protocols can be both insulting and demotivating. We also know from experience that even when the infrastructure and materials (described in behaviour change theories as the ‘opportunities’) to utilise knowledge are in place, this often fails to translate into hoped-for behaviour change. The reasons for this are highly complex. Michie et al. (2011) focus on the role that motivation plays in combining knowledge and opportunities to facilitate individual behaviour change. We felt concerned that many of these theories, rooted in psychology, tended to overemphasise the individual and gave insufficient attention to the structural contexts that crush motivation. Turning to ideas from evolutionary economics, we were captivated by the concept of ‘imagined realities’. As we framed it in the book: ‘Is it possible in the context within which Ugandan health workers are placed to imagine a different reality?’ (p. 103). We contend that only in situations where a critical mass of people can begin to imagine sustained positive change can effective knowledge mobilisation and behaviour change take place. Harding makes a similar point in relation to research methods arguing that feminist research is, ‘interested in models that stress context rather than isolated traits and behaviours. Interactive rather than linear relations, and democratic rather than authoritarian models of order’ (1991: 301). The emphasis on behaviour change needs to be at the level of the organisation and organisational culture and not specifically, at the individual.

This experience framed our approach to the MSI. And to that extent, it cannot be described as strictly ‘inductive’ as we are continually learning from prior knowledge and associated research review. We proposed to build on our existing strong relationships with managers and local health workers established through continuous engagement and strengthened through the deployment of local and international ‘volunteers’. We did not conceive of these K4C faculty (and ourselves) as the experts with solutions, but as colleagues playing a supportive role and engaging in mutual learning. We were fully aware that the expertise on the ground is of equal and often greater importance. Each of the participants, whatever their cadre or level of seniority, brings new knowledge, ideas, and enthusiasm to the table. Where specialist knowledge has been required, this has tended to take the form of multi-disciplinary encounters, of horizontal knowledge exchange with an emphasis on communication skills. Antimicrobial resistance is highly complex and, from a scientific perspective, conceptually difficult. Understanding the processes of culturing and testing bacteria for resistance and the implications of this for prescribing behaviour is not easy for those of us who are not trained in microbiology or pharmacy. Wanting to acquire such knowledge demands a real commitment and an ability to imagine a different approach to working. With this in mind, we designed an intervention based on K4C’s commitment to co-presence with continuous project engagement on the ground. This creates an environment within which carefully managed and planned short stays can have a high impact and bring new interest into a project. This applies not only to visiting colleagues from the UK but also to students and interns passing through the wards. Where the need and interest are articulated and the timing is right, short stays may provide important opportunities for more formal training input. Where possible, we have conducted this in ‘bite-size’ chunks on the ward. And where the opportunity has arisen and the demand was patent, we have provided this through larger multi-disciplinary formal training sessions. A British volunteer pharmacist observed the success of this approach and likened it to knowledge transfer mechanisms in the NHS:

A clear indicator of knowledge change is progress in the hospital and clear progress has been made. There is clear visibility of pharmacists on the ward – midwives know what to do with culture and sensitivity tests. The whole model is mirror like to the NHS. Clearly knowledge exchange has taken place we have just done it in an unconventional way on the ground; in a much more informal and effective way.

The following section presents interview data to illustrate the effectiveness of this approach from a variety of perspectives. We open with the views of the hospital administrator with whom K4C has had a strong relationship and who has played a key role in the partnership. Asked to outline what his experience of this short intervention has been, he immediately refers to the mentoring/co-working approach distinguishing it from other projects he has witnessed in the hospital:

The project has made strong inroads. Your approach is very involving. The people who are on the project are working together. They are part of it. It’s a real departure from many projects that we have seen where the people who come with the project go and then the project also goes. Where aliens are imposed on people; they come with their ideas and then they go. It’s very involving. The midwives feel part of it. For me it has been a very exciting project and very successful – we feel a part of it. There has been a real change in people’s perspectives about how they approach their practice. No little thing is ignored – the little things that we were ignoring - like you repair a tap – and yet we had the capacity to do these things, but we didn’t do them until the project came.

[Why that is the case?]

We had a laissez-faire kind of attitude when looking at institutional things – our consciousness has been awakened. Right now, they will say ‘hey can we work on this?’ The training that you have brought with the project – both the formal way - but you see with [K4C midwives] they have worked with the team with an in-built training mechanism that has strengthened our collaboration. They come on board and support changeovers of staff. We should be able to see change beyond the lifetime of the project. There seems to have been an integration of minds.

[What are the main benefits for you in administration?]

The project has helped our Human Resource by increasing numbers of staff on the wards and by improving staff training and guiding people on how sepsis is to be managed. The hospital now has the resource of more trained people. When they go, I still have trained staff.

The final part of the Administrator’s comment alludes to the augmentation of staffing that co-working brings. It raises the question of whether the benefits have come from simply having more staff time on the ward and will be extinguished as and when that disappears. Certainly, local staff welcomed the additional support. During the project, a new in-charge was placed on the ward, in a Ministry rotation from Kampala. It is clear from the following midwife’s narrative that the new in-charge had played a major role in instilling leadership by example and co-working herself:

Since those K4C people came in, they had to unite us. They just go and give good dressing. You find you are now working as a team. It is because of the teamwork those people introduced. They showed us good leadership. They would follow-up and afterwards they call the doctor and he comes. Even if they first deny we call again, we wait and again we call - even the senior doctors. The good leadership because when those people came in everyone was willing to act.

[Why wasn’t there some resistance to these people?]

The way how they smell the sepsis cases. We used to say ‘we are not going there’ but when those people came in, at first it was SMELLING but you find them really dressing wounds. They were working WITH US hand in hand. They taught us how to do it, so we gained knowledge and they are good leaders to us.

[Where did the leadership change come from?]

They were really working alongside us hands on. We saw them dressing wounds and the in-charge she dresses wounds too, so we are all working together now. She is the in-charge, but you find her dressing wounds. This was not happening before. Before the leader said, ‘I am the leader, so I am not dressing’.

The new in-charge also noticed the difference between hands-on co-working and formal training. When asked if running CMEs would have been as effective as having K4C midwives on the wards, she replies:

I prefer ‘on-the-spot-training.’ This is what has changed attitudes and the fact that the K4C team are ‘hands-on’ working as part of the team. We have had CMEs but they heed no results. They listen, hear, but do not implement. With CMEs there is often no follow up – you need constant follow up to change behaviour. And how [K4C IPC midwife] is on the wards at times watching them hand wash and pulling them up – not criticising but making them constantly aware of the importance of hand washing both to themselves and to the patients.

Another midwife suggested that there may be some rollback if the K4C staff are removed from the ward at the end of the project:

We would like it to stay that good. Maybe it won’t be 100% but maybe 50% because when they are there, they are additional. But [they] are not just numbers. When they are there, they know exactly what to do and you know that. ‘Hey, [she] is there – let me do this other part’. It is a division of labour and we work together. They know when I am on the ward then sepsis is covered!

It is interesting to note that the observation that the K4C midwives ‘know exactly what to do’ is not a reflection of greater initial knowledge or experience. None of the Ugandan K4C team had previously worked on AMR or sepsis management. They have, as many of the local staff have, grasped the opportunity to learn through co-working. An intern doctor expresses a view that, although local staff now have the knowledge, further support on the ward is needed to put it into practice:

The care these ladies (K4C midwives) are doing is making a big difference.

[But are the local staff also doing the same care or do they rely on K4C staff?]

They would do good but not as much as there is always inadequacy of staff. You may find only 1 or 2 nurses on the ward and lots of work to do so they won’t be able to dress the wounds…

[Is that because the local staff lack time or is it skill/knowledge?]

They have the skills but there are not enough staff on the ground. One nurse can’t give drugs, receive mums from theatre, manage septic wounds etc. It is not really knowledge – the big challenge is staff time but when we have nursing students around that pressure is down. Now it is holidays so that’s when we have issues.

Another intern doctor observed a benefit arising from having more staff on the wards which has enabled local midwives to ensure that medication (and antibiotics) is administered on time. Timely administration is extremely important to ensure the efficacy of treatment and reduce AMR:

In terms of my observations in post-natal and gynae, I see more timely medication now; they are still not receiving it on time as desired but there has been improvement. The K4C staff have taken over the gynae and peri sepsis cases and wound dressing and it is now done on time. The workload of the local staff has reduced so that they are now able to administer medication on time.

[Was only K4C midwives doing the wound dressing now then? Was it just a question of workload?]

Now – you see swabbing and samples going to the laboratory – doing a lot of following up is very hard for us to do so now [we have K4C midwives] – they will withdraw a sample, get it tested – get results in 3 days and change the prescription and you get the patient recovering so quickly.

[So, is it having more staff or knowledge?]

I think it’s a two-way thing. Let’s say if the MOH gave more staff. If you had 10 more staff, I don’t think they would take swabs and test them - they would go back to their old excuses. So, it’s not just a reduction in workload that brings the change. It is important to note that we need people employed specifically to do that – to fight infection on the ward – dedicated people. It means our people have not appreciated the importance of doing that.

I still think if we don’t have that they will go back to their old ways – sitting around pressing their phones and not doing their work. It could slide back if you removed Rachel and Dorothy. There is a solution to that. I don’t know if it is poor motivation or… you see even Dorothy and Rachel are not employed as usual nurses – I think if they were not here, they would not be doing the work they are doing. It’s because it is their designated job – they are employed by K4C to do this. We need to have people employed specifically for that.

At this point in the interview, he seems to suggest that the changes may lack sustainability. However, his perspective shifts as he refers to the inheritance of a ‘trait’ on the ward:

Wound management has improved greatly, and mothers are receiving twice daily dressings, so they improve so quickly. The staff have developed a trait to inherit. It’s good that the local staff have seen the others. They know if you have a wound and take good care of it, they will get better quicker. It’s a great impact. It is even something you can push into other facilities.

In response to a question about the engagement of local midwives, he explains how one of the local midwives*, ‘goes the extra mile to do better. She keeps reminding me*’. He then describes the improvement in team working and the impact this has had on his own behaviour:

Leadership on the ward – you see the medical profession is hierarchical – a system without leadership is bound to fail and I think it works now. Yes at one time Rachel did great work - I spoke rudely to her as she followed me and insisted I look at a patient who I had already passed by in the ward round – but they had the results of the tests back after I’d seen her – she kept pushing me. In the end the senior pharmacist prescribed.

He acknowledges that this pressure (from a midwife) had been a good thing in encouraging him to respond immediately to the results:

this has been a great thing for me as a doctor – now I have seen how significant the tests are and prescribing medicines on time I think I will use that as a skill even when I leave FPRRH.

These reflections are interesting. He attributes the behaviour change on the ward not simply to having more staff but what he perceives as the allocation of specialist, dedicated, roles to staff. In practice, this was not the case. K4C did not allocate designated roles or role descriptions to our colleagues. They were placed on the ward and, with the support of a UK (diaspora) nurse who had previously undertaken specialist work on surgical site infections and wound management, they collectively undertook a scoping exercise. This identified some key problem areas which they then started to work on with local staff to resolve. Nevertheless, his proposal that staff on the ward be given specific roles may be worth considering. His contention that it is mainly K4C midwives engaging in wound management and swabbing is not borne out by the interviews, or perhaps more importantly by ethnographic observation. Asked whether all staff or just K4C staff are engaged in these activities, a midwife comments:

We are all doing it and the laboratory is working. I believe we ALL know what to do now – we can take off the samples – arrange prescriptions – we can – we are able – we will continue – of course the main thing will now be staff shortages.

One of the pharmacists commented on the specific value of mentoring and hands-on co-working suggesting that it was not just the extra staff but the fact that they engage hands-on alongside the local staff:

Because there are K4C personnel on the ground working with people and mentoring them. I have a strong suspicion if they were not there, we would not have seen this change – showing people how to do things and mentoring them. They are hands-on and people see what they are doing – how they are cleaning wounds and people will follow their lead – they see them actually doing the work and feel motivated to join in. You see them criss-crossing up to the laboratory. If you don’t have people on the ground, you won’t get accurate information – I’ve noticed that, so presence on the ground is key.

[Would the same change have come if we had UK volunteers]

You need people on the ground for a long period. The perception if there are British people, they see whites as only coming for a short time. They are there but anyway they will go away so they would rather have a local person who stays longer.

[What about [K4C nurse volunteer]? She came for a short time?]

Well they saw her as local – she was accepted as a local. Even me I thought she was Ugandan.

As we noted above, the mentoring relationships on the ground are complex and fluid with knowledge exchange taking place in a variety of directions. The greater engagement of the pharmacists on the ward has created new opportunities for active mentoring within the pharmacy team itself. One of the senior pharmacists describes this process:

The interns are happy to do the clinical work now. It is challenging, motivating, and inspiring but they appreciate my presence and my authority on the wards. It has opened their eyes to the possibilities in their professions. Most pharmacists tend to be frustrated when they qualify and there is nothing for them to do in the hospital apart from signing requisition orders… our system does not support our role. It gives them confidence.

The decision, by the project team, to employ one of the pharmacy interns when his internship came to an end mid-project has stimulated a number of mentoring opportunities. In the first instance, it has freed up the hospital pharmacist to engage directly with staff, students and interns on the wards. Second, it has extended a period of more intense mentorship and enabled him to get more involved in leadership and research:

[He] was on his way out but he has benefitted from that – he has got what his colleagues didn’t get. Clinical pharmacy has to be practical; you can’t sit in the classroom. You have to have the practical relevance and you need someone who inspires and encourages you.

The expectation that projects such as this must involve formal, off-ward CMEs, was expressed by a senior pharmacist visiting the hospital on behalf of the Commonwealth Pharmacy Association who insisted on knowing what formal training the pharmacy interns had received. The senior hospital pharmacist replied:

We have had interactive, informal, training. The people on the ward now ask for the pharmacists – there is real behaviour change – they didn’t used to ask.

It is interesting to consider how the K4C staff reflected on their own roles on the ward and the impact of these. One of them used the word ‘ training’ when talking about local staff. This triggered a question about what that involved:

[You refer to them doing ‘what we trained them to do’. When did we train them as such?]

Well when I do a procedure, I work with one of the staff. We work together to do the procedure. Wound dressing is a two-person job, so we work side by side.

[How do you do that? If a wound needs dressing do you go and ask someone to help?]

Yes, I just go and ask someone to come and help me. Sometimes we assist them. It doesn’t have to be us doing it all the time. Most of the time you need an assistant, so you don’t contaminate things around you – so each time one person is doing the dressing and the other person is an assistant.

On rare occasions when there is only one staff on the ward you find you’re doing it yourself but like yesterday we found a nurse doing a wound, so I went along to help. She doesn’t see it as a threat, we work together. It’s the way people come on board that matters because if you come and they see you as one of them … but if you come at another level and they look at you as telling them what to do – the working relationship would be poor – me I see them looking at us to help. As long as were there to give a hand they will usually come on board.

As noted previously, the K4C team were not themselves experts when the project commenced. They describe how they have learnt through a process of mentoring within a multi-disciplinary team:

It is like a continuous update. When I was in (college) training, they did not go into details about taking samples. Now we have more knowledge about it, we know how we are doing it and we can interpret the results and see how to get these isolates causing this problem so somehow, we are getting knowledge.

[Did you have that knowledge at the start of the project?]

Not as such - somehow - but I know a lot more now about the microbiology

[So, how have you learnt then?]

Because we have multi-disciplinarity. If it is a new isolate, we can ask in the laboratory and the pharmacy. If the antibiotic is not working, we can look at alternatives. Then you get to know.

[So, you have learnt as a team but with them]

Yes, we get knowledge from different cadres – from the doctors – the laboratory – we have learnt together. And [lab scientists] trained us as a team on how to take cultures and how to interpret them – he trained us on the ward. They showed us how to take sterile swabs and avoid contamination - we have also not got any contamination in our swabs – according to the results from the laboratory.

Also, because they are changing antibiotics, that is a group thing, so we learn as a team. We discuss it. We never find just a pharmacist changing the drug – we all discuss it together. Like when we discussed a patient reacting to doxycycline, we all discussed it together. So, it is a multidisciplinary team.

[In the past would you have started to ask such questions about antibiotics?]

No, but as soon as it started it was a multi -disciplinary approach.

When you get this patient on the ward, we pull everyone together and discussion takes place. People realise it is not a one-man job. It is more interesting as most of the time the interns are on the ward, so everyone realises this is something we have to do. Ourselves, this has been new to us. Now there is a lot coming through – now we see the results of teamwork. Everyone is concerned about a patient wasting away and this has motivated everyone.

## Knowledge Transfer and Behaviour Change

Harding’s work on objectivity in research describes how development policies targeted at the Global South are imbued with concepts of knowledge deficit and, as a result, failed to achieve their objectives. The ‘strong objectivity’ approach, on the other hand, she suggests has been more effective:

The transfer of Western scientific rationality and technical expertise from the West to “the rest” had always been the “motor” of modernisation theory and now drives development policy […] many of the assumptions about women and poor people in the Global South - were false..[..] the strong objectivity approach enabled the goals of improving to the living conditions for poor women and of decreasing poverty overall to be advanced… (2015: 152)

We have noted in previous work (Ackers and Ackers-Johnson 2016) the tendency of international development funders to assume an immediate relationship between behaviour change and knowledge. Describing this as an example of ‘fetishizing training’, we illustrated the complexity of apparently simple interventions (in that case operationalising triage) and the associated behavioural change required. Antimicrobial resistance is a far more complex concept than triage. Little was known about AMR until very recently and the language used prohibits multi-professional engagement. Although the science is complex and still emerging, the necessity of achieving effective science communication is paramount. It has been inspirational to hear local midwives articulate questions about AMR in sophisticated scientific terms:

Then of course with the culture and sensitivity – right now we are doing OK. You have guided us on that one.

[Did you know how to do it before?]

Not really. We knew how to take swabs, but we didn’t know how to interpret the results. I have really begun appreciating; now we know which organisms we are fighting and can give the required antibiotics.

[So, have you had to learn a lot about these organisms and the antibiotics?]

Yes, we have had to learn a lot. The major thing I’ve learnt is about how mothers here are resistant to most antibiotics. Before I would hear but I had not seen it myself but at least now I have observed. Now there is a case who is only sensitive to amikacin.

## The Contribution of Formal Training in a Mentoring Context

In keeping with an inductive approach, the MSI did not commence with formal training or efforts to introduce externally produced tools or protocols but with on the ground relationship-building and co-working. Towards the end of the project, we have organised more formal training usually in a small ‘doctors’ room on the ward or in the larger hospital boardroom. Semi-formal short CMEs on topics such as hand hygiene or management of the newly organised evacuation room (and associated cleaning and sterilising procedures) have been bolted onto to clinical audit meetings which, since the project, occur every time there is a maternal death or a complex case for discussion. During a project visit in January 2020, the team responded immediately to requests from local midwives and intern doctors to improve their ability to interpret laboratory results and provided a short ward-based CME. Strictly speaking, midwives don’t ‘need’ this knowledge; their role in theory is to handover the results to the doctors and for pharmacists and doctors to deliberate and prescribe. The desire to know more and understand the processes which had led to such remarkable improvements in patient outcomes is an indicator of the motivational impact of change and how that fosters a quest for knowledge.

Somekh (with reference to educational learning in the UK) does not use the language of behaviour change that has become so popular in recent years but links learning closely to notions of personal efficacy and autonomy: ‘learning is closely related to a sense of personal efficacy – to learn children need to have autonomy and take responsibility’ (2006: 4).

This has echoes of the idea of imagined realities and the belief that things can change, and you can be a part of that change. Defining ‘agency’ as the ‘capability of a self to take actions that will have an impact on a social situation’, Somekh contends that change processes need to, ‘*unlock the agency of individuals and groups so that groups work interactively and reflectively to go beyond their personal learning and aim for a broader impact on improving working methods and practice across the whole workplace’* (2006: 21).

Of course, a sense of personal agency is inextricably contingent on the context (although we would argue that there is always at least some potential for agency). Whilst behavioural scientists may use the more benign concept of ‘opportunities’ to capture context, social scientists have articulated this relationship in terms of structuration theory or structure-agency models that account in more complex ways for the webs of constraints that shape individual behaviour.

The final chapter sums up the achievements of the Maternal Sepsis Intervention approach with a discussion on how you capture change processes in complex interventions; how you sustain those changes and how lessons can be applied to other settings.

## Footnotes

1 https://hms.jhpiego.org/wp-content/uploads/2016/08/LDHF_briefer.pdf

2 Rather than rehearsing the theoretical arguments, here we refer you to Chapter 4 in our book, ‘Can (imported) knowledge change systems? Understanding the dynamics of behaviour change’ (pp. 79–113) available as a free download at—Mobile Professional Voluntarism and International Development: Killing Me Softly? (2017–Open Access)
